# Supplementary material for: Reduced North Pacific Deep Water formation across the Northern Hemisphere Glaciation
Source: Nat Commun. 2025 Mar 19;16:2704. doi: 10.1038/s41467-025-58069-x (PMC11923177; doi:10.1038/s41467-025-58069-x)
Supplement: Supplementary file 1 — Supplementary Information [file 41467_2025_58069_MOESM1_ESM.pdf]

Supplementary Information

For PSUSolver the following equations are used:

1. Mg/Ca = 0.9 + (0.1 x BWT) (from ref [1], with the correction to account for reductive cleaning process). An analytical uncertainty of ± 0.085mmol/mol (2σ) was used.

2. BWT = 15.75 - (4.46 x ($\delta^{18}O$ - $\delta^{18}O_{sw}$ + 0.27)) (from ref [2], the equation relating BWT and $\delta^{18}O_{sw}$ to benthic $\delta^{18}O$ values from *Cibicidoides wuellerstorfi*). An analytical uncertainty of ± 0.05‰ (2σ) was used.

Supplementary Figures

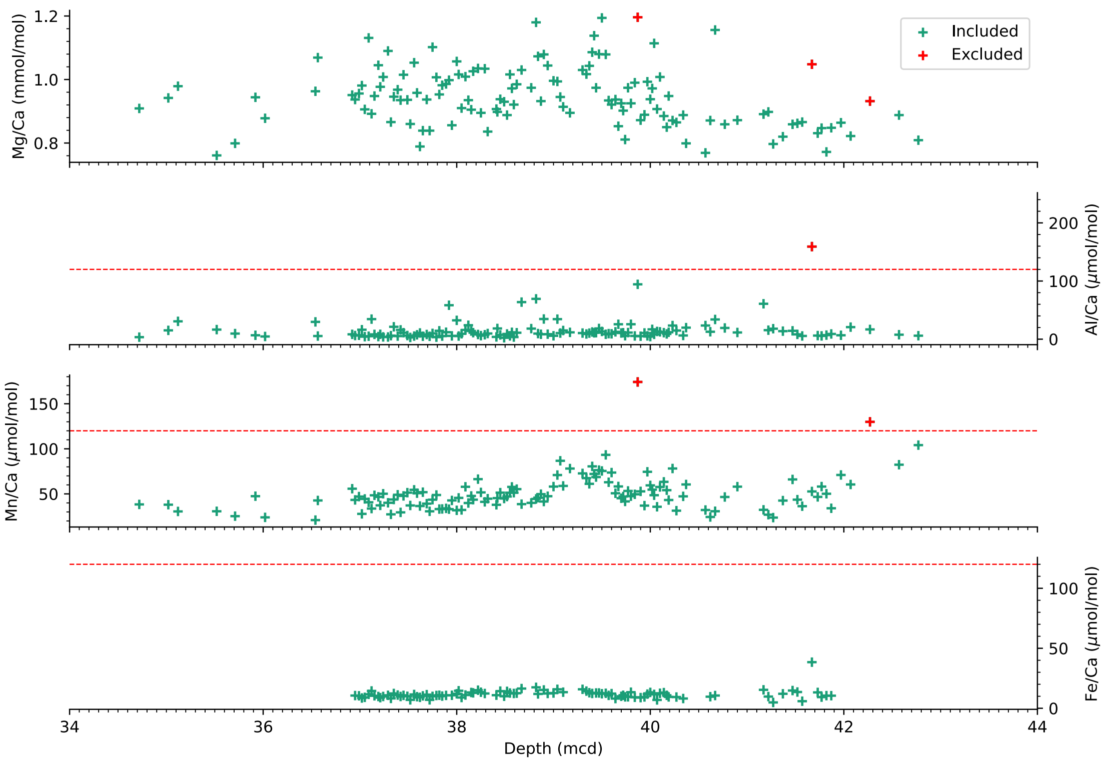

Supplementary Fig. 1 Contaminant trace metal ratios for Site 1209.

Measured Mn/Ca, Al/Ca, and Fe/Ca ratios against depth from Site ODP 1209. Samples with values of Mn/Ca, Fe/Ca, or Al/Ca above 120 µmol/mol were excluded from subsequent analysis and are in red.

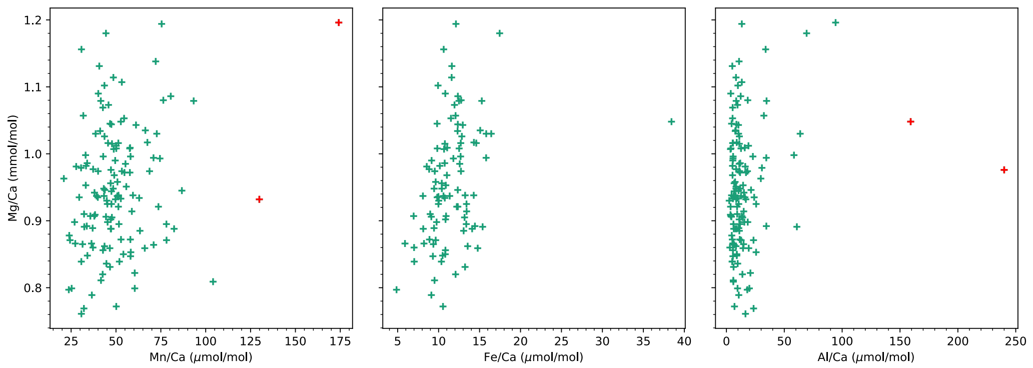

Supplementary Fig. 2. Cross plots of contaminant trace metals against Mg/Ca for Site 1209.

Cross plots of measured Mg/Ca against Mn/Ca, Al/Ca, and Fe/Ca from Site ODP 1209. Samples with values of Mn/Ca, Fe/Ca, or Al/Ca above 120 µmol/mol were excluded from subsequent analysis and are in red.

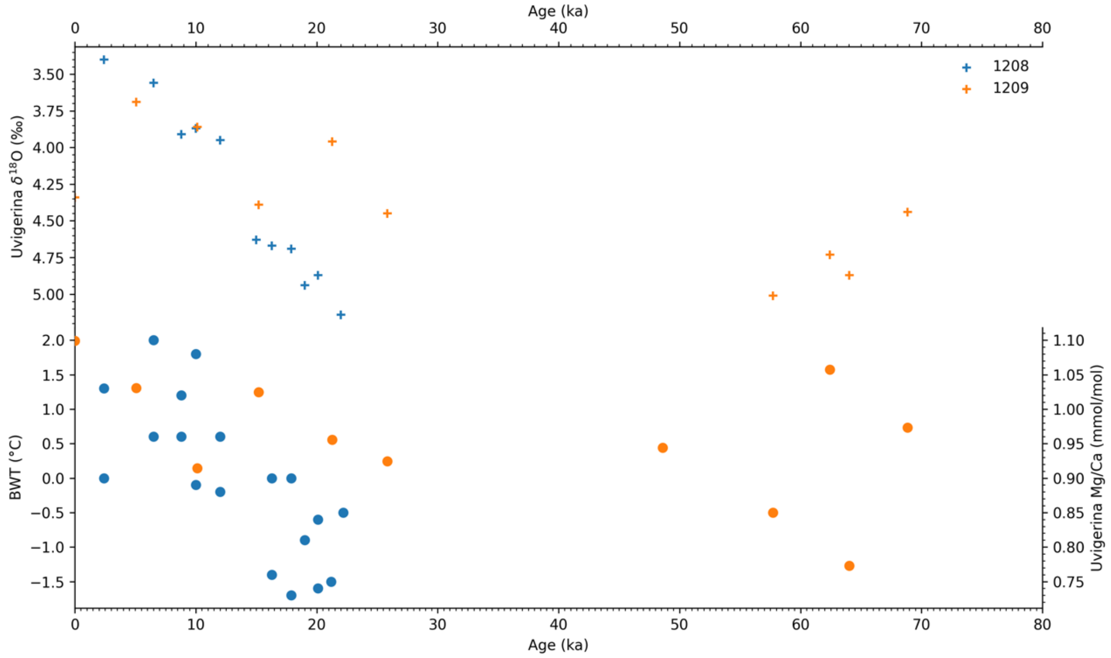

Supplementary Fig. 3. Core top comparison of benthic δ^{18}O and Mg/Ca values.

(a) Core top values of benthic δ^{18}O from *Uvigerina peregrina* showing no distinct difference in δ^{18}O values between ODP 1208[3] (blue crosses) and ODP 1209[4] (orange crosses), or slightly more positive δ^{18}O values at Site 1208 contrary to what is seen in the Late Pliocene. The difference in benthic δ^{18}O

presented in the rest of the paper is between $\delta^{18}O$ values from *Cibicidoides wuellerstorfi*, but as Extended Data Figure 8 shows, the same difference in oxygen isotopes appears in the *Uvigerina* record. (b) Core top values of Mg/Ca from ODP Sites 1209 (orange circles) and 1208[3] (blue circles) showing colder temperatures at the deeper site 1208 contrary to the Late Pliocene. Mg/Ca values were converted to bottom water temperatures using the formula from ref [1] shown above.

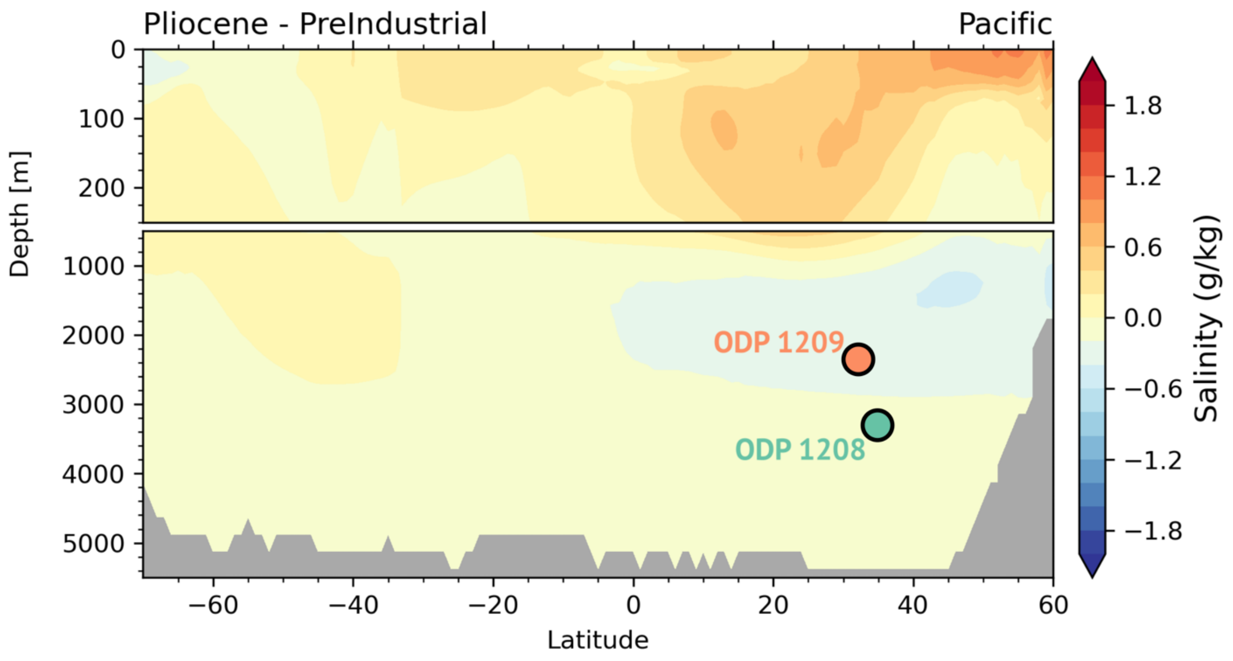

Supplementary Fig. 4. Salinity model output for the Late Pliocene.

Model output from ref[9] showing salinity in the deep Pacific Ocean in the Late Pliocene relative to a preindustrial. Location of Site 1209 (orange) and 1208 (blue) are shown as circles.

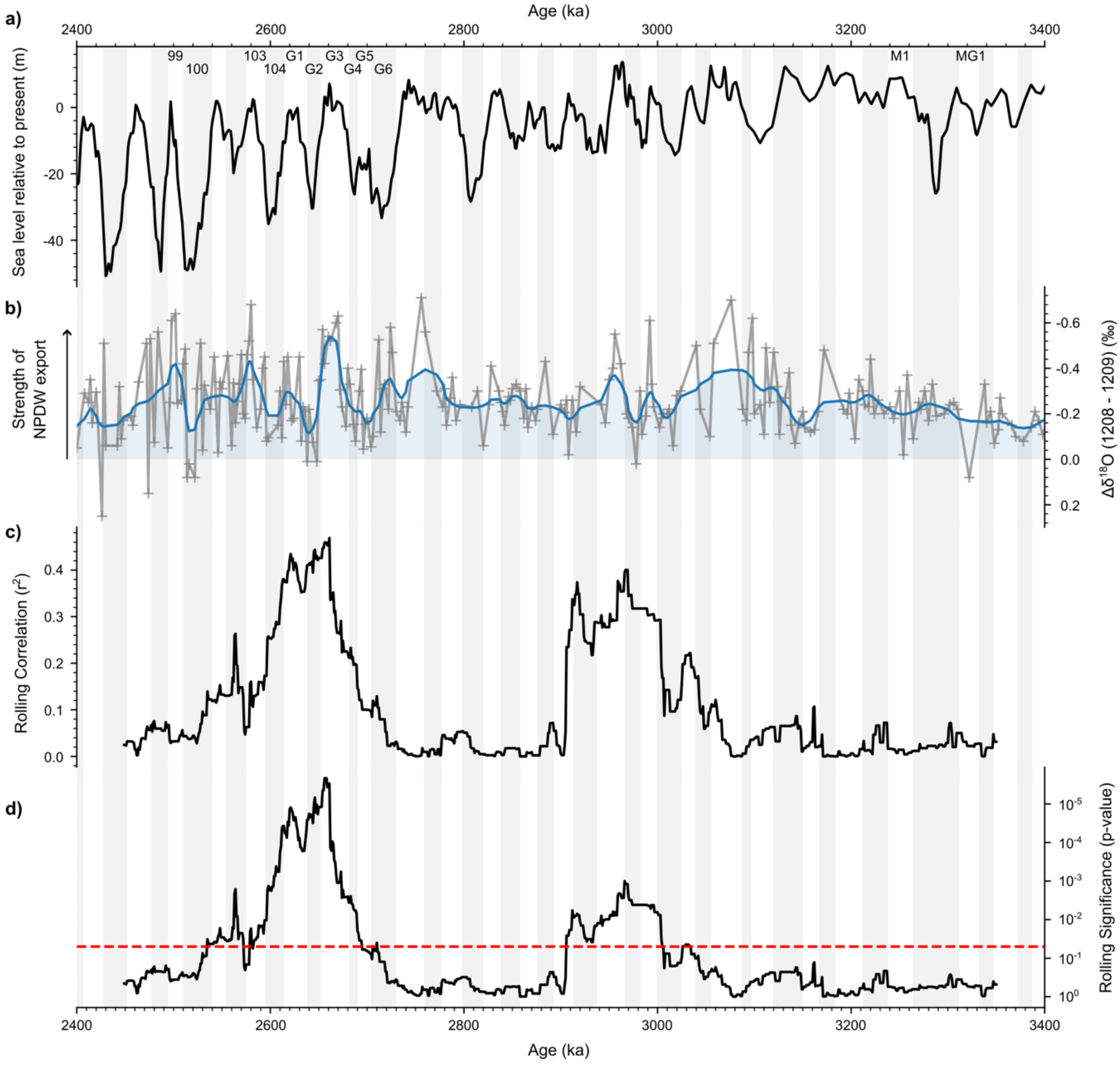

Supplementary Fig. 5. Correlation between the difference in benthic $\delta^{18}O$ values of Site 1208 and 1209 and global sea levels.

(a) Sea level curve showing past sea levels relative to modern in metres[5], with an age model tuned to LR04[6]. (b) The difference between the benthic isotope $\delta^{18}O$ records (1208 - 1209) run through a 5-ka second order Butterworth low-pass filter (pink) and the unfiltered differences (grey crosses) for the period 3.4 - 2.4 Ma. (c) Correlation calculated using a Pearson's Correlation test (r^2) between the sea level curve and the difference in the benthic isotope $\delta^{18}O$ records (1208 - 1209) on a rolling 100-ka window. There is a strong correlation between the sea level curve and the difference in oxygen isotopes after MIS G6, as well as around KM5 (3.1 Ma). (d) Significance calculated using a Pearson correlation test (p-value) between the sea level curve and the difference in the benthic isotope $\delta^{18}O$ records (1208 - 1209) on a rolling 100-ka window. Areas above the red line have a p-value of less than 0.05, suggesting that if sea level and the difference in oxygen isotopes were unconnected post MIS G6, it would be

unlikely to see this amount of agreement. There are also p-values less than 0.05 for the period following MIS KM5 (3.1 Ma), though this is during a time of elevated sea levels and thus unlikely to be due to closure of ocean gateways. Glacial marine isotope stages according to the LR04[6] age model are highlighted and annotated.

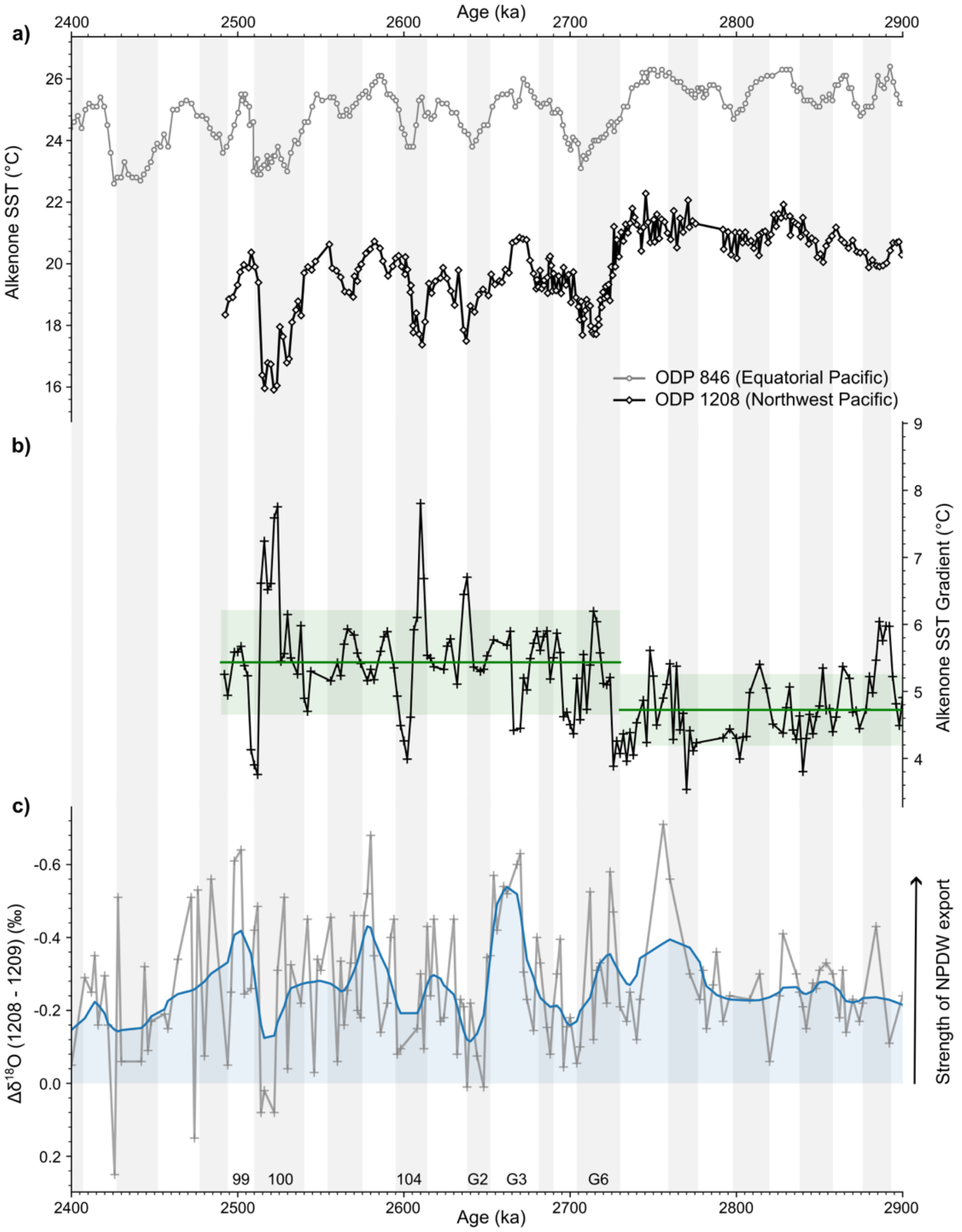

Supplementary Fig. 6. Change in the Pacific SST Gradient over the iNHG.

(a) Alkenone derived sea surface temperatures (SST) from ODP Site 846[7] (grey circles) in the Equatorial Pacific and ODP Site 1208[8] (black diamonds) in the Northwest Pacific Ocean. (b) The difference between the two alkenone SST estimates (846 - 1208). The average value of the SST gradient (green)

and the 1σ standard deviation are shown for before and after MIS G6. Before 2.73 Ma, the average SST gradient is 4.72 ± 0.54°C, after 2.73 Ma the average SST gradient is 5.43 ± 0.78°C. (c) The difference in benthic oxygen isotope values between ODP Site 1209 and 1208 (grey) run through a 5-ka second order Butterworth low-pass filter (blue). A positive difference indicates more positive $\delta^{18}O$ values at Site 1208. Glacial marine isotope stages (according to the LR04[6] age model) are highlighted. The difference in benthic $\delta^{18}O$ values seems to be inversely correlated with SST gradient after MIS G6 (2.73 Ma).

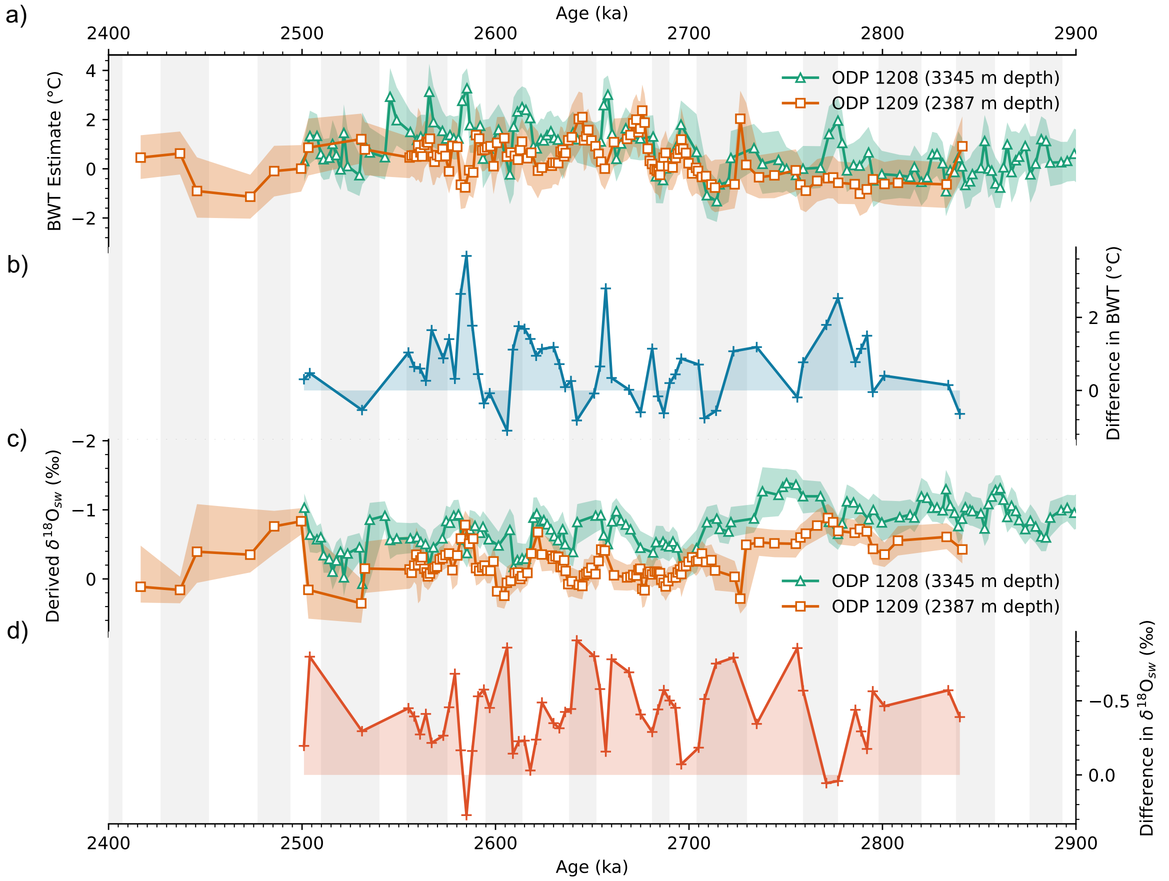

Supplementary Fig. 7. Difference in BWT and $\delta^{18}O_{sw}$ estimates.

(a) BWT estimates from Mg/Ca measurements from Site 1208[3] (green triangles) and 1209 (orange squares) for the period 2.4 – 2.9 Ma. (b) The difference in BWT between the two sites. This is calculated from a resampled record of the two sites resampled to the mean BWT value over a 3-ka window. (c) Derived $\delta^{18}O_{sw}$ record from Mg/Ca and $\delta^{18}O_{benthic}$ measurements from Site 1208[3] (green triangles) and 1209 (orange squares). (d) The difference in $\delta^{18}O_{sw}$ between the two sites. This is calculated from a resampled record of the two sites resampled to the mean $\delta^{18}O_{sw}$ value over a 3-ka window. Orange and

green shading indicates 95% confidence interval. Glacial marine isotope stages according to the LR04[26] age model are highlighted and annotated.

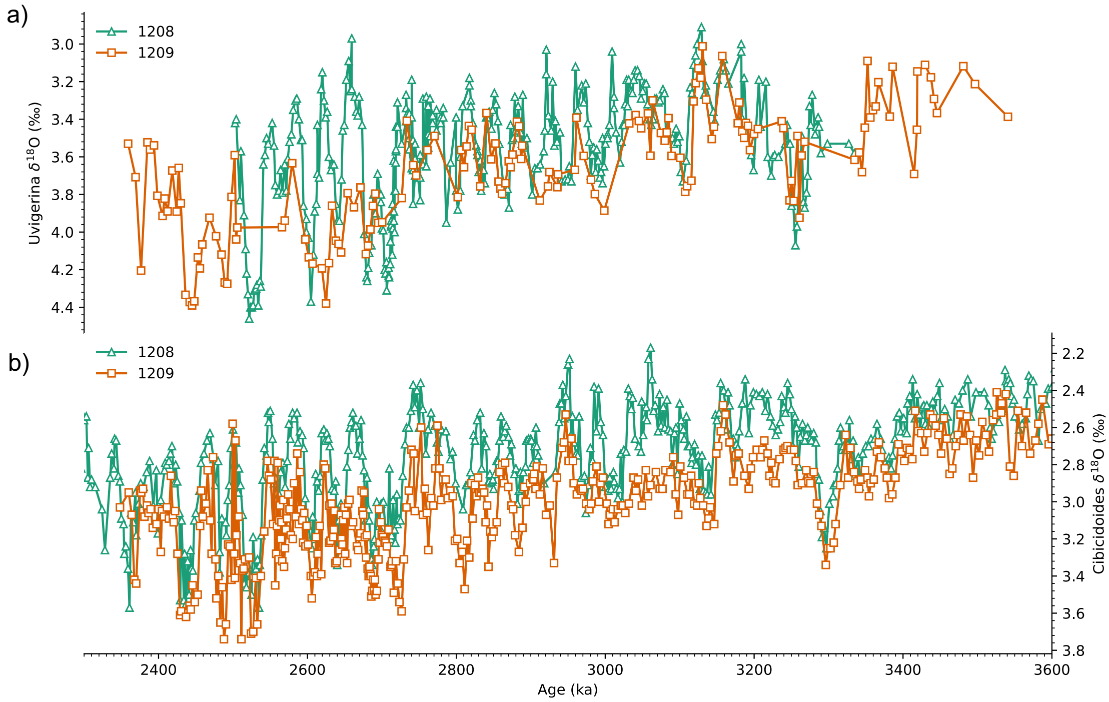

Supplementary Fig. 8. Comparison of Uvigerina and Cibicidoides $\Delta\delta^{18}O_{1208-1209}$.

(a) Benthic isotope δ^{18}O records from ODP Site 1209[9] (orange squares) and 1208[3] (green triangles) from benthic foraminifera species *Uvigerina* spp. (b) Benthic isotope δ^{18}O records from ODP Site 1209 (orange squares) and 1208[10] (green triangles) from benthic foraminifera species *Cibicidoides wuellerstorfi* showing that both species record the same signal of heavier oxygen isotopes at the shallower Site 1209 (2387 m depth) compared to the deeper Site 1208 (3346 m depth). The higher resolution of the *Cibicidoides* record is why this record is used for this paper.

Supplementary Tables

Dataset	Kolmogorov-Smirnov test		Shapiro-Wilk test		n
	Statistic	p-value	Statistic	p-value	
1209 $\delta^{18}O$	0.985	0	0.991	2.75e-09	421
1208 $\delta^{18}O$	0.985	0	0.977	0.0124	727
1209 Mg/Ca	0.247	1.59e-07	0.989	**0.3995***	131
1208 Mg/Ca	0.154	3.06e-05	0.985	0.0095	231

Supplementary Table 1. Statistical Tests to show the non-normal distribution of $\delta^{18}O$ and Mg/Ca data from Sites 1208 and 1209.

*The BWT data for site 1209 cannot be conclusively said to not be normally distributed according to the Shapiro-Wilk test. However, the very low p-value with the KS test comparing the dataset to a normal distribution suggests that it is very likely not normally distributed.

Supplementary References

1. Elderfield, H. *et al.* Evolution of Ocean Temperature and Ice Volume Through the Mid-Pleistocene Climate Transition. *Science* **337**, 704–709 (2012).
2. Marchitto, T. M. *et al.* Improved oxygen isotope temperature calibrations for cosmopolitan benthic foraminifera. *Geochim. Cosmochim. Acta* **130**, 1–11 (2014).
3. Woodard, S. C. *et al.* Antarctic role in Northern Hemisphere glaciation. *Science* **346**, 847–851 (2014).
4. Bordiga, M. *et al.* Calcareous plankton and geochemistry from the ODP site 1209B in the NW Pacific Ocean (Shatsky Rise): New data to interpret calcite dissolution and paleoproductivity changes of the last 450 ka. *Palaeogeogr. Palaeoclimatol. Palaeoecol.* **371**, 93–108 (2013).
5. Rohling, E. J. *et al.* Sea level and deep-sea temperature reconstructions suggest quasi-stable states and critical transitions over the past 40 million years. *Sci. Adv.* **7**, eabf5326 (2021).
6. Lisiecki, L. E. & Raymo, M. E. A Pliocene-Pleistocene stack of 57 globally distributed benthic δ^{18}O records: PLIOCENE-PLEISTOCENE BENTHIC STACK. *Paleoceanography* **20**, 1–17 (2005).
7. Herbert, T. D. *et al.* Late Miocene global cooling and the rise of modern ecosystems. *Nat. Geosci.* **9**, 843–847 (2016).
8. Martínez-García, A. *et al.* Iron Fertilization of the Subantarctic Ocean During the Last Ice Age. *Science* **343**, 1347–1350 (2014).
9. Ford, H. L. *et al.* Sustained mid-Pliocene warmth led to deep water formation in the North Pacific. *Nat. Geosci.* **15**, 658–663 (2022).
10. Venti, N. L. & Billups, K. Stable-isotope stratigraphy of the Pliocene–Pleistocene climate transition in the northwestern subtropical Pacific. *Palaeogeogr. Palaeoclimatol. Palaeoecol.* **326–328**, 54–65 (2012).
